# Child protective services involvement and exclusionary school discipline

**DOI:** 10.1111/cdev.13941

**Published:** 2023-05-10

**Authors:** Sarah A. Font, Reeve Kennedy, Tenesha Littleton

**Affiliations:** 1 Pennsylvania State University, State College, Pennsylvania, USA; 2 East Carolina University, Greenville, North Carolina, USA; 3 University of Alabama, Tuscaloosa, Alabama, USA

## Abstract

The study examined the impact of child protective services (CPS) contact on out-of-school suspensions for 49,918 Wisconsin students (followed from ages 5–6 to 14–15; [school years 2010–2019; 74% White; 7% Black; 11% Hispanic; 8% other; 49% female]). A quasi-experimental design comparing recent CPS contact to upcoming (future) CPS contact shows that both recent CPS contact without foster care and future CPS contact predict higher odds of suspension compared with no contact. Higher odds of suspension emerged prior to CPS contact and did not substantially increase during or after CPS contact, suggesting that system-induced stress is not a primary driver of behavioral problems leading to suspension. Foster care reduced the odds of suspension among White children and children in special education.

Child maltreatment is associated with adverse behavioral health outcomes, including rule-breaking and aggressive behavior in the home, school, and community. Yet, there are also concerns that the primary system tasked with intervening in cases of child maltreatment—child protective services (CPS)—can lead to or worsen behavioral problems, due to the stress or fear induced by investigations and potential removal from one's family of origin (Dettlaff et al., 2020; Roberts, 2021). Alternatively, CPS involvement may improve children's behavioral health by engaging parents in interventions that address harmful parental behaviors (e.g., substance abuse, domestic violence) (Baron & Gross, 2022) and by facilitating access to behavioral or mental health treatments for the child, either in the home or in foster care.

Children involved with CPS, including children in foster care, tend to have poor school outcomes, including lower attendance, test scores, grades, and higher rates of disciplinary events resulting in suspension or expulsion (Berger et al., 2015; Scherr, 2007). Poor school outcomes for children involved with CPS are consistent with established developmental impacts of child abuse and neglect (Font & Kennedy, 2022). However, it remains unclear whether and through what mechanisms CPS contact, in particular, increases or decreases adverse school outcomes. In this study, we follow a 2010 cohort of nearly 50,000 kindergarteners for 9 years to assess how the risk for out-of-school suspension differs before, during, and after CPS contact.

We focus on exposure to suspensions rather than other educational outcomes for three reasons. First, studies using appropriate counterfactual conditions show a consensus that foster care is unlikely to reduce, and may modestly improve, academic achievement and engagement (Bald et al., 2019; Berger et al., 2015; Font & Maguire-Jack, 2013; Gross & Baron, 2022). In contrast, there is far less consensus on how foster care affects health and behavior (Berger et al., 2009; Sariaslan et al., 2022) or involvement with institutions that are tasked to respond to illicit or antisocial behavior, such as juvenile and criminal justice systems (Bald et al., 2019; Baron & Gross, 2022; Doyle Jr., 2007; Doyle Jr., 2008; Roberts, 2019). Second, there is widespread concern that contact with CPS is, itself, traumatic, even when no intervention occurs, but empirical evidence is lacking. To evaluate acute responses to stress, it is helpful to have longitudinal event-level data, such as suspensions. Whereas suspension occurs following a specific behavior or set of behaviors, other academic metrics, such as grades or standardized tests scores, reflect cumulative work or learning across a sustained period of time. Third, exclusion from school due to suspension can itself adversely impact school engagement and performance (Noltemeyer et al., 2015), and, for children facing difficult home circumstances, deprives them of a source of external support and resources.

## BACKGROUND

### Child maltreatment and behavior

Child maltreatment includes both acts of commission—physical, sexual, and emotional abuse—and acts of omission, or neglect. Neglect constitutes a majority of the circumstances that lead to CPS interventions, such as foster care, though neglect frequently co-occurs with abuse (Herrenkohl & Herrenkohl, 2007) and abuse may be under-documented in case files (Loomis et al., 2020). Decades of research, using a range of samples, methods, and measures, provide clear evidence that both abuse and neglect adversely affect children's development, though potentially through different mechanisms (Font & Kennedy, 2022).

Of particular relevance to schooling, child maltreatment is associated with cognitive and learning delays (Veltman & Browne, 2001), problems with attention and executive function (Masson et al., 2015), emotional dysregulation (Messman-Moore & Bhuptani, 2017), and anti-social behavior and other behavioral problems (Braga et al., 2018). Further, abusive and neglectful home environments often lack a consistent structure, schedule, and set of expectations. A lack of oversight and structure in the home environment may provide children with limited practice or skills with following instructions or routines (Manly et al., 2013)—a typical expectation in school. In addition, because neglect can involve limited social interaction or cognitive stimulation in early life, neglected children may be academically and social delayed (Manly et al., 2013; Spratt et al., 2012), resulting in frustration or disengagement in school activities or attention-seeking behavior. Finally, maltreated children may have witnessed or been subjected to violence or other antisocial behavior in their homes and communities and may learn and imitate those behaviors at school (Font & Kennedy, 2022). These exposures, individually and cumulatively, may increase the likelihood of school disciplinary problems.

Young children are highly susceptible to omissions in parental care because they are limited in their capacity to protect themselves or access outside assistance. Maltreatment in the first 3 years of life—a critical development period (Holmes et al., 2018)—may incur long-term damage to brain development (Harden et al., 2016) and position children for difficulties adjusting to school environments (Bell et al., 2018), which could predispose them to disciplinary action. Yet, abuse and (especially) neglect can be and often are chronic or recurrent experiences (Kim & Drake, 2019) and maltreatment that persists beyond early childhood is strongly associated with a range of adverse health and developmental outcomes (Font et al., 2022; Ireland et al., 2002).

### Effects of child protective services contact

Following a referral from a mandatory reporter or concerned party that involves a reasonable suspicion that a child has experienced or is at imminent risk of abuse or neglect, CPS initiates an investigation. School personnel account for about one in five CPS referrals nationally (U.S. Department of Health and Human Services, 2021) and comprise a large share of referrals for school-aged children. Most referrals result in no formal intervention or ongoing involvement with the family. Yet, the experience of a CPS investigation could be traumatic or harmful to children (Raz, 2020) due to being asked invasive questions, potentially experiencing a physical examination for injuries or indications of abuse, and the looming threat or actuality of removal from the home. Children may also fear causing trouble for their parents or being removed from their homes, even if they are being abused or neglected (Wilson et al., 2020). Studies of children subjected to investigations, including physical examinations of abuse, suggest low levels of distress, particularly when best practices are applied (Jones et al., 2007; Lazebnik et al., 1994).

When children are at imminent risk of serious harm, invasive or upsetting questions or examinations may still be necessary and preferable to the alternative if it facilitates risk-mitigating intervention. Yet, given the low rate and quality of interventions (Berger & Font, 2015; Jonson-Reid et al., 2017), CPS investigations may seed false hope. Children who disclose abuse or neglect to CPS may not be believed or taken seriously (Font et al., 2020; Wilson et al., 2020); they often receive no supportive services or intervention and continue to accrue new harms following the investigation (Helton et al., 2022). Such experiences may evoke disappointment, hopelessness, or anger as children realize that nothing is changing. Although children of all ages may be harmed or benefited by CPS intervention (in-home or out-of-home services), the potential direct harms of CPS investigation (exposure to invasive questioning or procedures; fear or unmet expectations regarding the outcome of the investigation) are most relevant for children who are, on the basis of age and development, able to be interviewed, describe their experiences, and grasp the potential implications of CPS contact. Thus, adverse effects of CPS investigations should be concentrated among children old enough to attend school, the focal time period for this study.

Because CPS contact is frequently used in research as an indicator of maltreatment, extraordinarily little research exists on the impacts of contact with CPS separate from exposure to child maltreatment and related family concerns that result in CPS referrals (Font & Kennedy, 2022; McKenna et al., 2021). One exception was a retrospective study of Canadian adults self-reporting childhood abuse, which found that adults who also reported CPS contact had similar or slightly worse mental health outcomes (Afifi et al., 2018). Yet, that study did not differentiate by level of contact (e.g., investigation only, foster care), and adults with a single CPS contact resulting in no intervention may be less likely to recall that event on a survey than adults with recurrent contact or an intensive intervention. Further, that study focused only on abuse, which constitutes a minority of US CPS investigations.

### Foster care

Foster care provides temporary homes for children who are found unable to reside safely with their families of origin. Because it is an intervention most often used following abuse or neglect where imminent risk of further harm remains, children frequently enter foster care with behavioral issues and unmet mental health needs (McMillen et al., 2005). Whether and how foster care impacts children's behavior remains disputed. Separation from one's parents, and sometimes siblings and other close relationships, can be highly traumatic (Mitchell, 2018) and thus may induce or exacerbate stress-related emotional outbursts and other psychological harms. Even in optimal circumstances—a safe, supportive, and stable foster home—the unpredictability that is inherent to foster care can take its toll on children (Font & Palmer, 2022). Beyond distress associated with the removal (Folman, 1998) and the uncertainty inherent to temporary care, adverse experiences within foster care—unstable, abusive, or neglectful placements and placements in group and institutional settings—may create and exacerbate behavioral problems (McFarlane, 2018). Adverse placement experiences are concentrated among older children and children with pre-existing behavioral problems (Konijn et al., 2019; Palmer et al., 2020), for whom states recruit fewer high-quality family foster homes (Government Accountability Office, 2015). Thus, foster care may be less beneficial, or more harmful, in adolescence compared with early and middle childhood.

Placement in foster care also has the potential to positively impact children's behavior. Children are most often removed from homes that involve—if not physical or sexual abuse—substantial parenting concerns, such as substance dependency and severe mental illness (U.S. Department of Health and Human Services, 2020; U.S. General Accounting Office, 1998). Thus—and not discounting the distressful experience of removal—children's cognitive and behavior functioning may improve if placed in a foster home that is safe, with sober and psychologically healthy caregivers who are able to ensure basic needs are met. Children in foster care also receive more health and education services than other children who received Medicaid (Harman et al., 2000) and these services typically relate to conditions or needs that preceded their entry to foster care (McMillen et al., 2005), implying unmet health care needs prior to removal. When the services provided, which may include therapeutic and pharmacological treatments for the child as well as behavioral health education and skills training for caregivers, are needed and effective, children's capacity to comply with school rules and structure may improve.

The effects of foster care are difficult to empirically evaluate. As an intervention of last resort, entry to foster care is highly selective and the primary factors that influence the decision to place a child in foster care—perceived risk and severity of harm—are difficult to quantify and observe. There is little agreement among studies on the impacts of foster care on various dimensions of antisocial or problem behavior (Baron & Gross, 2022; Berger et al., 2009; Doyle Jr., 2007). This may reflect heterogeneous impacts of foster care, which depend heavily on particularities of agency policy and practice, as well as the degree of risk if left at home. The experience of foster care varies widely across place and time in terms of length of stay, types and stability of placement, and the quality of services and supports.

### Disruptive behavior and disciplinary events

Exclusionary school discipline is associated with reduced academic achievement and increased likelihood of school dropout (Noltemeyer et al., 2015) and juvenile and criminal justice systems involvement (Cho et al., 2019; Davison et al., 2022). These negative associations partly reflect selection bias: children with more severe behavioral issues are suspended and expelled at higher rates and also face greater risk for poor educational and justice system outcomes. However, rigorous studies find tentative evidence that exclusionary discipline has a negative causal effect on justice system involvement (Bacher-Hicks et al., 2019) and effects may be concentrated among the most disadvantaged students (Mittleman, 2018).

Positive or negative behavioral changes following child maltreatment, CPS contact, or foster care placement may induce changes in exposure to suspension. However, there is a complex association between individual behavior and school discipline. Education personnel, such as administrators and teachers, have some discretion in the use and form of disciplinary actions. The degree of discretion is greater for less severe conduct that violates school rules and may be minimal for offenses that constitute criminal acts, such as those involving violence, weapons, or drugs (Welsh & Little, 2018). A majority of suspensions result from less severe offenses (Skiba et al., 2014). Reflecting varying levels of both student misconduct and use of punitive responses to misconduct, suspension rates vary across classrooms, grade levels, schools, and districts and, within those units, by race, gender, and disability status (Welsh & Little, 2018).

The same school personnel who refer students for disciplinary action and determine the severity of punishment are a primary source of referrals to CPS among school-age children. Thus, they are often aware of students' home situations, including whether they are in foster care. This information may induce sympathy for the child, with educators interpreting behaviors as an expression of stress or trauma, rather than willful disobedience or disrespectfulness. Alternatively, knowing a child is in foster care may result in stigma, whereby educators apply negative stereotypes to the child (Wildeman et al., 2017), such that typical child behavior is pathologized and catastrophized. In either case, Black and male students may be most impacted by the (positive or negative) labeling effects of foster care given that they already experience higher levels of suspension for subjective offenses (Welsh & Little, 2018).

## CURRENT STUDY

In sum, CPS may be a source of stress or support, depending both on the extent of risk in their home environment and on the quality and appropriateness of CPS response or intervention. Yet, because CPS encounters children during difficult family circumstances, traditional approaches to analysis may conflate the effects of the family circumstances that *lead to* CPS involvement with the effects of CPS contact itself. This study leverages a unique dataset to assess two questions: (1) how is CPS contact associated with out-of-school suspension for disciplinary infractions?; and (2) how do associations differ depending on the intensity of CPS contact (no placement vs. foster care)? We use various identification strategies to assess whether associations are likely causal in nature, assess heterogeneity by grade level, special education status, sex, and racial-ethnic category, and possible mechanisms underlying these associations. Given that prior research would support conflicting hypotheses (spanning positive, negative and null effects of CPS contact and foster care placement), we offer no a priori hypotheses and position this study as semi-exploratory.

## METHOD

### Data and sample

Data for this study were drawn from the Wisconsin Administrative Data Core (WADC), a linked administrative database maintained by the Institute for Research on Poverty at the University of Wisconsin-Madison. The WADC contains records from various public systems: this study specifically leveraged data from the Department of Public Instruction (DPI; education) and the Department of Children and Families (CPS records). The target sample was all Wisconsin children who entered kindergarten at a DPI school in the 2010–2011 school year (*N* = 61,454). Approximately 85% of WI children attend DPI schools (vs. choice, private, or home schools) at any given time, but students may not remain consistently at a DPI school. For study inclusion, children must have been enrolled in a DPI school in each school year from 2010–2011 through 2018–2019 (grade 8, unless held back; *N* = 50,014). Requirement for ongoing enrollment excluded the expelled students who re-enrolled outside the DPI system following expulsion or spent a full year non-enrolled. For consistency, we also excluded students who were expelled but remained a DPI student in each year, leaving an analytic sample of 49,918.

#### Education records

For each of the 9 years of observation, students had one observation per month for September through June (typical span of academic year) if enrolled in that month. Where students changed districts during a month, they were identified as enrolled in the district in which they spent the majority of time. The primary outcome of interest for this study was out-of-school suspension. For the study cohort, there were 21,888 suspensions involving 6553 individual students. Supplemental outcomes include the reason for suspension, which was collapsed into two groups. *Severe infractions* were suspension events that occurred due to assault, endangering others, possession or use of drugs or alcohol, or bringing a weapon to school. This category accounted for 41% of all suspensions; of these, the overwhelming majority were categorized as assault or endangering behavior. *Rule violations* reflect an umbrella category of “other violations of school rules”, accounting for 59% of all suspensions observed in the data. There was no additional detail available on the nature of those violations, but they would broadly include infractions such as dress-code violations, cheating, or plagiarism, verbal bullying or harassment, or disruptive or disrespectful behavior.

Additional measures drawn from DPI records were grade level (kindergarten through 8th), child demographics (race, ethnicity, and sex), and prior school-year homelessness, free or reduced lunch enrollment, and disability status for special education services. We used prior year indicators because foster care may move children out of homelessness or lead to an evaluation for conditions that confer special education eligibility. Foster care also confers near-automatic eligibility for free or reduced lunch (95% of recent foster care observations had free or reduced lunch enrollment in the current school year). Because homelessness, free or reduced lunch program, and disability status are available only at the school-year level, we cannot fully determine temporal ordering within school year. Disability type is the primary condition that makes a child eligible for special education services. The included categories were as follows: none, autism, speech and language, learning disability, intellectual disability or traumatic brain injury, physical impairment, emotional and behavioral disorders, significant developmental delay, and other health impairment. Other health impairment includes a broad range of chronic or acute health conditions (e.g., ADHD, asthma, leukemia, and diabetes) that can negatively impact school performance. We cannot discern whether special education students are placed in separate classrooms for all or part of their school day, where behavioral expectations and disciplinary guidelines may differ.

District characteristics were created from the individual-level student files and are constructed within each school year. Specific measures were racial and ethnic composition (percent Black, percent Hispanic), rate of suspension and expulsion, rate of student homelessness, and rate of student enrollment in free or reduced lunch program. Of note, school-level variables or identifiers were not included in the dataset and we cannot assess heterogeneity in characteristics, including use of suspension, within school districts.

#### Child protective services records

CPS records are based on the Wisconsin State-Administered Child Welfare Information System (WISACWIS) and include dates of both CPS investigations (regardless of substantiation or intervention decision) and foster care (FC) placements. Records were available at (or before, for larger counties) July 2004, covering over 99% of children from birth through the end of the study period, with 100% coverage for ages 1 year and onward. CPS contact prior to kindergarten (birth until Fall 2010) was categorized into five groups (1) no known CPS contact; (2) one CPS investigation, no placement (NP); (3) multiple CPS investigations, NP; (4) FC to reunification; (5) FC to adoption; and (6) FC to other permanency or remained in care. These groups are broadly consistent with prior research indicating that prior CPS contact is strongly predictive of future contact (Kim & Drake, 2019) and may also inform the extent or severity of intervention (use of FC) upon subsequent contact. However, the link between prior and future victimization or intervention is unlikely to hold for children placed in adoption or guardianship due to the change in caregivers (who are the most common perpetrators).

For time-varying CPS contact from the start of kindergarten (September 2010) through the end of 2019, we first calculated a running measure of recent CPS contact, defined as contact within the past 6 months (e.g., a child with CPS contact in April of 2012 would have a value of 1 for each month from November 2011–April 2012). Type of contact is divided into two groups: CPS investigation with no foster care placement (CPS-NP; their families may or may not receive formal or informal in-home services) and foster care placement (CPS-FC). A child with an investigation and FC in the same month is categorized in the CPS-FC group, as placement constitutes the more intensive form of CPS contact.

We then identify children who are not recently or currently involved with CPS (no investigation or placement in the past 6 months) but (will) have CPS contact—either an investigation or removal to foster care—within the *next* 6 months. This group is referred to as Future CPS and functions as a quasi-experimental condition used to study program effects in various fields (Berger et al., 2015; Fenelon et al., 2022). Where a child has both past 6 months and next 6 months CPS, they are assigned to ‘past six months’ group. Approximately 13.8% of children with recent or current CPS-NP and 8.2% of children with recent or current CPS-FC experience a new CPS investigation or a removal in the next 6 months.

Supplemental measures categorize CPS investigations by report source to assess whether children face differential risk of suspension when educational personnel were the source of allegations about (and thus aware of) the child's potential abuse or neglect victimization. Lastly, because children may remain in care for a long period of time and our primary measures do not differentiate among a child who was recently removed, a child who recently exited, and a child in longer-term foster care, we construct an additional measure that separates out new removals to FC, recent exits from FC (predominantly re-unifications or relative guardianships), and ongoing FC episode (in FC continuously for the past 6 months). This is not our primary measure due to its complexity and because the fine-grained parsing of the sample results in a loss of statistical power.

### Analytic approach

The data were organized by student and month, with month running from September 2010 (beginning of kindergarten) through June 2019. We excluded the months of July and August of each year, when students are typically not in school and thus not at risk for suspension. For students with enrollment gaps within a given year, any month in which they were not actively enrolled was excluded. Because students who experience suspension have lower attendance on average and because children in foster care may experience enrollment gaps when they move schools, excluding incompletely enrolled students would reduce the representativeness of the sample on core experiences of interest. However, the recent and future CPS contact measures refer to the past or subsequent 6 months of time and incorporate CPS events that occurred over the summer or when children were not enrolled. The panel variable is the student, and the time variable is the month of observation, where September 2010 is equal to 0 and June 2019 (the last month of possible observation) is equal to 105. In total, there were over 4.2 million months observed for the 49,918 students. Across the 9-year observation period (including summers), there were 11,288 CPS investigations pertaining to 6388 unique children and 16,760 months of foster care involving 1039 children.

We considered two types of models for longitudinal data, each offering different strengths and limitations. All models were produced in Stata 17. First, we employed random effects logistic regression with robust standard errors using *xtlogit*: these models provide a variance parameter (sigma) and a statistic (rho) that characterizes the proportion of the total variance attributed to the panel variable. In the main models, rho exceeded 0.5, suggesting that the majority of variation in suspension was explained by the student. Random effects models include strong assumptions—namely, that the panel variance is uncorrelated with the covariates. This assumption is violated in our models. Thus, in addition, we consider child-fixed effects logistic regressions: these models require weaker assumptions but force the exclusion of students who are never subject to suspension or CPS contact. That is, the individual fixed effects models narrowly consider the subset of students who experience one or more suspensions (13.1% of the sample) and address the question of how changes in CPS contact are associated with changes in the odds of suspension among those who experience a change at some point during the observation period. Thus, fixed effects models findings may not generalize. We present both sets of models, as well as a series of sensitivity analyses, to characterize a possible range of effects.

All models include grade-level fixed effects. The probability of suspension increases nonlinearly by grade and is concentrated in grades 7 and 8. We use grade rather than age because, though they are highly correlated, behavioral expectations in classrooms likely follow students' grade level rather than their age. We also include an indicator for children who are old for their grade level (e.g., age 7 in kindergarten; age 8 in first grade). All models also include calendar month fixed effects because suspensions are not evenly distributed throughout the school year. Lastly, models included either current school district fixed effects or a set of school year-district characteristics (school-specific indicators are not available). Although district fixed effects are a stronger strategy for eliminating confounding bias (e.g., overlap between CPS contact rates and suspension rates that stem from unobserved dimensions of institutional policy or culture), they force exclusion of all children attending school in a district that did not suspend any sample members. As such, they induce a tradeoff between internal and external validity concerns. We present both models to assess consistency of findings.

Although the various analytic strategies described above reduce bias in our estimates, they do not allow us to discern whether effects of CPS contact are plausibly causal. Thus, our identification strategy rests on using future CPS contact among children with no recent or current contact as an appropriate counterfactual or comparison condition for current or recent CPS contact. Children who are living in “high-risk” circumstances that will soon result –but has not recently resulted—in CPS contact can be compared with children who have recent or current CPS contact. (In the child fixed effects specification, children are compared to past and future versions of themselves). The premise of this comparison is that the “effect” of CPS contact on any child well-being outcome is, at least in part, artificially inflated by selection bias. That is, child maltreatment affects both CPS contact and various child outcomes, but the presence, nature, or severity of child maltreatment is not observable in this (or many other) studies independent from CPS contact. Thus, a direct comparison of children with and without current CPS contact conflates any effects of CPS contact with the established effects of maltreatment. Similarly, even within-child comparisons of periods with and without CPS contact may be biased if their environments change—including onset or desistance of maltreatment—in the lead up to or longer-run after-math of CPS contact. By considering risk of suspension in the immediate lead-up to CPS contact, we can approximate the “effect” of maltreatment and related adversity that leads to CPS contact separately from the “effect” of CPS contact itself. If we observe that the odds of suspension are higher in periods of current or recent CPS contact than in periods immediately preceding CPS contact, we would tentatively conclude that CPS contact increased the risk of suspension beyond the effects of family circumstances that resulted in CPS contact.

Our results proceed as follows: (Table 1) sample description; (Table 2) full sample estimates of the three main models (random effects, random effects plus school district fixed effects; child fixed effects); (Table 3) subgroup estimates by grade level groups (K-4th, 5th-6th, 7th-8th) and special education status; (Table 4) subgroup estimates by sex and race; and (Table 5) supplemental analyses of CPS report source and detailed FC entry and exit status. The Supplemental Appendix includes: (A) collapsed estimates of overall CPS contact (any recent compared with future); (B) linear probability estimates for all models in Table 2; (C) Cumulative rates of CPS contact and suspension within subgroups presented in Tables 3 and 4; and (D) models separately estimating suspension by reason (severe infractions vs. other rule violations).

## RESULTS

### Sample description

Table 1 displays descriptive statistics for the full cohort and the subgroups with any CPS contact in kindergarten or later and any suspension (column percentages). The full cohort was approximately 74% White, 7% Black, and 11% Hispanic, with small numbers of all other racial and ethnic groups. Black children were overrepresented in the CPS, CPS-FC, and suspension subgroups, with the largest degree of over-representation for suspensions. Males constitute 69% of all children experiencing suspensions. We note that gender identity was not available in our data. Other relevant sociocultural attributes were too infrequently observed to allow for meaningful assessment (e.g., children born outside the U.S.).

Approximately 10.5% of the sample had one or more CPS investigations prior to Kindergarten: 5.7% had a single CPS investigation without foster care, 2.9% had multiple CPS investigations without foster care, 1% entered foster care and reunified, and less than 1% entered foster care and were adopted or remained in foster care at the outset of kindergarten. However, of those who would experience FC in kindergarten or later, most (65%) had prior CPS contact. Thirteen percent of child school-year observations pertained to a child who received special education services in the previous year due to disability, with speech and language disabilities comprising the largest share of qualifying disabilities. Approximately 1.2% of child school-year observations involved past-year homelessness, and 36% involved past year free or reduced lunch eligibility. Children who had CPS contact or experienced foster care during the observation period were more likely to be in special education, to experience homelessness, and to receive free or reduced lunch.

### Regression results

All of the main models focus on comparison across four conditions, which we reiterate and abbreviate here to avoid redundancy: (1) no investigations or foster care in the prior or upcoming 6 months, “No CPS”; (2) recent CPS contact but no placements in foster care, “CPS-NP”; (3) recent or current foster care placement, “CPS-FC”; (4) upcoming investigation or removal, but no current or recent CPS, “Future CPS”. Results of the main regression models are displayed in Table 2. Coefficients are in exponentiated form: adjusted odds ratios (ORs).

Model 1 shows that CPS-NP is associated with 78% higher odds of suspension than No CPS. Future CPS also predicts 60% higher odds of suspension, and coefficients for Future CPS and CPS-NP were statistically equivalent. In contrast, CPS-FC was not statistically significantly associated with suspension compared with No CPS and predicted lower odds of suspension than either CPS-NP or Future CPS. In short, children faced statistically equivalent odds of suspension in the 6 months following a CPS contact than before that CPS contact, and if they were in foster care, they faced lower risk than before. Results were similar in direction, relative magnitude, and statistical significance for M2, which added district fixed effects, and M3, the within-person fixed effects model. Results were also consistent with linear probability estimates (Appendix B).

Although not a primary focus of this study, M1 and M2 also show that early life (pre-kindergarten) CPS contact was a consistent predictor of suspension, especially CPS contact indicative of more severe or chronic maltreatment.

#### Subgroup models by grade level and special education placement

See Appendix C for rates of CPS, CPS-FC, and suspension by subgroup. Table 3 presents models separated by grade levels that roughly correspond to elementary (K-4th grade), intermediate (5th-6th grade), and junior high (7th to 8th grade) phases, and by current special education status. In these models, we sought to account for variations in behavioral expectations by grade level and special education status. Overall, we find similar patterns of association between CPS contact and suspension in each grade grouping. However, Future CPS may be a weaker signal for suspension risk in the junior high group versus the younger grade levels.

Among children currently in special education, the odds of suspension were statistically equivalent for CPS-FC and No CPS conditions, and CPS-FC was associated with significantly lower odds of suspension than children with CPS-NP or Future CPS. Yet, for children not currently in special education, CPS-FC predicted higher odds of suspension at a similar magnitude as CPS-NP and Future CPS.

#### Heterogeneity by sex and racial-ethnic category

We share the findings of subgroup analyses by sex and race to illuminate disparities and advance discussions about racial equity within systems. We caution that assessment of racial disparities in the effects of CPS contact is problematic given that our outcome—suspension—is itself subject to racial bias at the individual and school levels (Barrett et al., 2021). Over half (51.7%) of Black students were suspended during the observation period, compared with 8.5% of white students and 17.6% of Hispanic students. Smaller disparities sex were also present: 18% of males and 8% of females experienced suspension (see Appendix C). Nevertheless, supplementary models using the random effects (M2) and fixed effects specifications (M3) and sub-grouped by sex and race or ethnicity are shown in Table 4.

The pattern of findings is similar across sexes, though the confidence intervals suggest more precise estimates for males (consistent with their higher rates of suspension) than females. For race and ethnicity, we only have adequate sample size to examine White, Black, and Hispanic students but caution that—due to group sizes, the Black and Hispanic subgroup models are less precise (wider confidence intervals) than the White subgroup model. The models of White students mirror the full sample models, showing statistically equivalent odds of suspension for CPS-FC, and higher odds for CPS-NP and Future CPS, compared with No CPS. For Black students, both CPS-NP and Future CPS were associated with higher odds of suspension. The CPS-FC coefficient was positive in both models (indicating higher risk for suspension than No CPS), but only reached statistical significance in M2 (random effects) and CPS-FC was not significantly different from CPS-NP or Future CPS in either model. For Hispanic students, confidence intervals were very wide, and patterns were somewhat difficult to discern. Although CPS-NP was associated with increased odds of suspension relative to no CPS, other coefficients were inconsistent in direction, magnitude, or significance across models.

#### Report source specificity

We sought to assess the potential for educators' knowledge of students' possible maltreatment exposure to result in differential odds of suspension (greater leniency or punitiveness). We presume that school personnel are aware of a student's potential victimization if a CPS referral was initiated by school personnel, especially in grades K-4th, where students tend to have a single teacher for most core subjects. In models differentiating CPS-NP by the source of the allegations, children had significantly higher odds of suspension if their recent CPS contact was initiated by education personnel versus any other source. This provides tentative evidence against the hypothesis of sympathy-induced leniency, and possible support for the hypothesis of stigma-induced punitiveness.

#### Foster care entry and exit

The second set of models in Table 5 consider a more detailed model of foster care. These models show that the protective effect of CPS-FC is concentrated among children who have been in care at least 6 months, potentially reflecting the time needed for children to access and benefit from supportive services (e.g., mental health care) and adjust to the change in home environment. Of note, findings also suggest that the effects may not persist after exiting FC,

#### Reason for suspension

Districts exercise substantial discretion in their use of discipline. We posit that rule violations permit more discretion than severe infractions (violence, drugs, weapons) and thus assess whether associations between CPS contact and suspension are driven by high- or low-discretion events. Appendix D shows that CPS-NP, CPS-FC and Future CPS were all associated with increased odds of suspension for a severe infraction, with no statistically significant differences between the three groups. However, the point estimates for children in foster care trended toward lower odds and were not significantly different from No CPS in the fixed effects model (M3), consistent with the main models presented in Table 2. Also consistent with the main models, CPS-FC predicted lower odds of suspension due to rule violations compared with CPS-NP or Future CPS.

## DISCUSSION

An abundance of evidence indicates that child maltreatment, both abuse and neglect, compromise children's cognitive, social, and behavioral development in ways that inhibit educational engagement and achievement (Font & Berger, 2015; Manly et al., 2013; McGuire & Jackson, 2018; Veltman & Browne, 2001). Yet, there is little consensus about the impact of CPS contact on children's development, in part because of methodological challenges in differentiating the impacts of child maltreatment from the impacts of the CPS response to maltreatment (Font & Kennedy, 2022). Our study used a statewide longitudinal database from Wisconsin to examine patterns of suspension for children involved with CPS due to suspected maltreatment and to differentiate the effects of maltreatment and related adversities that lead to CPS contact from the effects of having contact with CPS. We highlight three findings.

First, children who encounter CPS experience high overall rates of suspension relative to the general population of students. Children with CPS contact before entering kindergarten were overrepresented among children experiencing suspensions, highlighting how childhood trauma may compromise children's long-term behavioral and socioemotional functioning and set a course for early and repeated exclusion from school. In addition, more than a third of children who had CPS contact after starting kindergarten had at least one suspension event over the nine-year observation window, compared with 13% of children in the full sample. Given that suspension is a response to misbehavior and behavior problems are a well-documented consequence of child maltreatment (Font & Kennedy, 2022), this is unsurprising. Nevertheless, exclusion from school is particularly problematic for CPS-involved children because they are less likely to have safe and stable home environments and suspension can provoke or accelerate disengagement and underperformance (Noltemeyer et al., 2015). Despite increased attention to the impact of trauma on students and related training and guidance for educators, we found no evidence that children's risk of suspension decreases when educators are aware of children's difficult home environments (i.e., are the origin of their CPS referral).

Second, we find no evidence that recent or current CPS contact without foster care has average positive or negative effects in the full sample or in any evaluated subgroup—at least in the short term – on children's risk for suspension. Ongoing concerns about the nature and reach of CPS intervention—in particular, that it may traumatize children more so than protect them—have been difficult to assess empirically because the measurement of child maltreatment is confounded with the CPS investigation and intervention. By leveraging our detailed longitudinal data, we compared the risk of suspension for children named as suspected victims on a recent CPS investigation versus children who will soon be investigated as a suspected maltreatment victim (i.e., are potentially exposed to maltreatment but that maltreatment has not recently been investigated). If the adverse outcomes of children with CPS contact were due to CPS itself, not the circumstances that CPS was investigating, then children would be expected to face higher risk of suspension during and after CPS contact versus before. Instead, we found that risk of suspension was statistically equivalent before and after CPS contact, and the risk for suspension was consistently higher compared with children who had neither recent nor upcoming CPS contact. In sum, the lack of change in suspension risk before and after CPS contact indicates that CPS contact, on average, appears to have little “added” short-term benefit or harm beyond the maltreatment concerns that evoke CPS contact.

Third, the nature of CPS contact matters. There is a clear conceptual difference in the experience of a CPS investigation, which may involve little more than a 15-minute conversation with a caseworker, and the experience of foster care, which can last months or (less often) years and entails a change in one's primary caregivers and physical environment. We found that recent foster care predicted lower odds of suspension in the full sample compared with both recent non-foster care CPS contact and any upcoming CPS contact and statistically equivalent odds compared with children with no recent or upcoming CPS contact. This pattern was broadly consistent across grade levels—though potentially less so for junior high students—and by sex. Weaker effects of foster care for older students is consistent with prior research demonstrating higher rates of mental and behavioral health problems (Palmer et al., 2022), a lack of qualified foster homes (Government Accountability Office, 2015), and increased rates of congregate care placement (Palmer et al., 2020) and placement moves (Konijn et al., 2019). By race and ethnicity, we found that differences between foster care and the other CPS conditions were typically not statistically significant for Black and Hispanic children, reflecting both different point estimates and wider confidence intervals due to smaller sample size. However, in no subgroup model was recent or current foster care associated with higher odds of suspension than recent CPS without placement or future CPS.

The protective effect of foster care was highly concentrated among children in special education. It is possible that special education and foster care contact jointly bring oversight and accountability that discourages suspension. That is, federal disability laws are designed to protect students from being penalized for the symptoms of their disability, but in practice, protecting students with disabilities from suspension may require advocacy by parties with institutional power and knowledge. The involvement of foster care caseworkers, as well as Court-Appointed Special Advocates or other professionals, may increase compliance with existing disability laws. Alternatively, students with disabilities, due to their higher levels of need, may disproportionately benefit from the increased resources, supervision, and health care services that are provided in foster care. CPS-involved children have high rates of inadequately detected and untreated physical, developmental, and mental health problems (Carr et al., 2020; Crozier & Barth, 2005), and—despite real concerns about the quality and consistency of therapeutic services (Szilagyi et al., 2015)—children in foster care are more likely than their in-home counterparts to receive assessment and treatment. This may also explain why, in our study, the benefits of foster care were concentrated among White children, as studies have consistently found that Black children receive fewer mental health services, even in foster care, irrespective of their need (Garland et al., 2003; Leslie et al., 2004; McMillen et al., 2004).

Supplemental analyses found that the pattern of findings was directionally similar for rule violations and severe infractions, though the protective effect of foster care (relative to CPS with no FC placement or future CPS) was larger and met statistical significance only in the rule violation model. Schools may give special consideration or leniency to children in foster care when the disciplinary action is most subjective (rule violations). However, if that were the case, it would be surprising for that consideration to not extend to children recently referred to CPS by educators—where it is most likely that the disciplinarian was aware of the child's familial hardships. Rather than leniency induced by sympathy, it may simply be that children in FC have more advocates with institutional knowledge (caseworkers, trained foster parents, service providers) who ensure that they receive supports and accommodations to which they are entitled.

Taken as a whole, our findings suggest that the “protective effect” of foster care for suspension may, in part, reflect that targeted compensatory supports, advocacy, and oversight are disproportionately provided to children in foster care versus children left in their homes following CPS contact (Berger & Font, 2015). However, other factors associated with foster care, such as reduced exposure to violence or increased structure or academic support in their home environment, may also benefit students. Further research is needed to understand and address potential gaps in the quality of foster care experiences for older children, children who are not in special education, and racial and ethnic minority children.

### Limitations

We note several limitations of this study. First, we use data from a single state and our sample does not include charter schools or private schools, which could reduce generalizability. Wisconsin children have a relatively low rate of early and middle-childhood CPS contact compared with other states (Kim & Drake, 2019); differences that may reflect lower rates of child maltreatment or higher thresholds for CPS contact. If the latter, then the population of children with CPS contact in WI may experience more severe or chronic forms of maltreatment than CPS-involved children elsewhere.

Second, although our identification strategy helps to differentiate the effects of CPS contact from the effects of circumstances that lead to CPS contact, we caution that our estimates are limited to short-term impacts (within 6 months). Relatedly, 10% of children had CPS contact prior to kindergarten but we cannot draw any conclusions about the positive or negative impacts of early-life CPS contact. We included indicators for earlier CPS contact as statistical controls, but their coefficients aggregate the effects of CPS contact with the antecedent maltreatment-related concerns and thus offer no insight on possible causal effects.

Third, foster care includes a wide range of settings and durations with varying quality of caregivers, service providers, and permanency outcomes. Because our models reduce this variability into a single indicator, we can speak only to average effects but acknowledge the likelihood that foster care confers both positive and negative effects depending on the child and their foster care environment (Font & Kennedy, 2022). In particular, we reiterate calls for further research on potential disparate impacts of foster care by race (Barth et al., 2020). Our study found that the protective effects of foster care were concentrated among White children, and Black children with CPS contact remained at heightened risk of suspension regardless of foster care placement. There are numerous possible explanations for this finding that cannot be evaluated in this study. We specifically note that Wisconsin's nonurban areas are overwhelmingly White and its urban areas are heavily segregated by race (Iceland et al., 2002). Thus, Black, White, and Hispanic children are exposed to distinct environments for school (Owens, 2018), and likely also different foster homes, service providers, and CPS agencies. These different institutional environments (school and CPS) may confer different benefits and harms. Our data only have district (not school) identification codes and limited information on CPS agencies practices and procedures. We also lack information on mental and behavioral health treatments and assessments that may illuminate disparate uptake or provision of services.

Fourth, it was impossible to fully discern whether differential patterns of suspension were primarily driven by differences in student behavior or differences in the responses to student behavior. District fixed effects address some confounding biases, but we could not account for differences within district in how schools respond to behavior issues. Given racial and economic segregation within districts, this is a meaningful limitation. School behavior, and especially suspension, is a narrow metric of psychosocial functioning and replication studies that incorporate other behavioral outcomes are needed.

Lastly, child fixed effects models cannot account for time-variant child-level confounding variables that may affect both CPS contact and student behavior. We rely heavily on our counterfactual of future CPS contact to provide an estimate of the potential rate of suspension that would be observed for children living in high-risk environments without CPS contact, allowing us to estimate the value or harm added by CPS contact as the difference between those who have CPS contact and those who do not currently but will have CPS contact in the near future. However, neither the past nor future measures provide a clear indication of the effect of maltreatment or associated family adversities given that children exposed to actual or suspected maltreatment vary from their peers on a range of economic, social, and environmental confounders that are also likely to affect well-being. Moreover, because some forms of maltreatment are unlikely to be reported to or investigated by CPS (e.g., emotional maltreatment) or do not fall under the jurisdiction of CPS (e.g., most nonfamilial sexual abuse), children with no CPS contact may nevertheless have experienced maltreatment, resulting in comparison group contamination (Shenk et al., 2016).

## IMPLICATIONS AND CONCLUSION

The findings of the current study highlight how maltreatment compromises children's school experiences and the limited role that CPS plays in mitigating adverse outcomes. CPS contact appears to be a meaningful signal of risk for adverse child outcomes, regardless of whether there is sufficient evidence or harm to warrant CPS intervention. Given the limited scope and resources of CPS, children who experience abuse or neglect typically receive no direct services from CPS (Berger & Font, 2015) and the services that may be provided to parents tend to be short-term and of limited impact (U.S. Department of Health and Human Services, 2021; Jonson-Reid et al., 2017). To improve outcomes for children who have, or are suspected to have been, maltreated, it is important that other institutions interfacing with children support them. Our public schools can do more to keep CPS-involved children in class and learning without compromising the safety or educational experiences of other children. Programs that seek to improve teacher skills in responding to student behaviors (more so than changing the behaviors directly) are most effective (Valdebenito et al., 2018). Incorporating a trauma-informed approach to these trainings could also provide teachers with a better understanding for how to meet the needs of maltreatment-exposed, as well as CPS-involved, children—as maltreatment and related experiences can have a substantial impact on cognitive and behavioral development (Bell et al., 2018; Veltman & Browne, 2001), thus impacting school readiness and the ability to comply with school rules and expectations. Lastly, restorative justice or similar strategies that emphasize empathy and relationship-building (Valdebenito et al., 2018; Welsh & Little, 2018) have shown promise in reducing suspension and improving school climate. These may be especially effective with maltreated children, who may have less opportunity to observe and practice those social-emotional skills with appropriate role models.

## Supplementary Material

supplement

## Figures and Tables

**Table 1:** Sample description (column percentages).

	Full sample	Any CPS, kindergarten onward	Any FC, kindergarten onward	Any suspension
*N*	49,918	6388	1039	6553
Child-level characteristics				
Any suspension	13.11	36.21	44.34	—
Sex				
Female	48.60	49.62	47.26	30.55
Male	51.40	50.38	52.74	69.45
Race and ethnicity				
White	74.23	59.99	54.19	48.79
Black	7.09	15.92	19.83	27.68
Hispanic	11.02	14.14	11.84	14.76
Native American	1.24	2.60	4.72	2.40
Asian/Pacific Islander	3.56	2.40	1.54	1.21
Multiracial	2.86	4.96	7.89	5.17
Highest level of CPS contact prior to kindergarten				
None	89.64	62.16	35.42	73.22
1 CPS report, no placement (NP)	5.65	17.44	17.71	12.93
Multiple CPS reports, NP	2.85	13.74	18.58	8.62
FC to reunification	0.87	3.98	7.51	2.61
FC to adoption	0.35	0.52	0.77	0.72
FC to other	0.64	2.16	20.02	1.91
Child-prior school year variables				
Past year disability status for special education				
None	87.13	73.48	67.31	73.33
Autism	1.27	1.76	1.15	2.26
Learning disability	2.91	5.35	5.02	4.27
Speech & language	3.61	4.75	5.36	3.78
Intellectual disability/traumatic brain injury	0.78	2.05	1.90	1.17
Emotional behavioral	1.37	5.81	10.59	7.83
Physical impairment	0.32	0.54	0.42	0.39
Significant developmental delay	0.27	0.67	0.96	0.51
Other	2.33	5.58	7.28	6.47
Past year homelessness				
No	98.83	95.10	92.29	96.14
Yes	1.17	4.90	7.71	3.86
Past year free or reduced lunch				
No	61.79	23.86	18.47	31.96
Yes	38.21	76.14	81.53	68.04

Abbreviations: CPS, child protective services; FC, foster care; NP, no foster care placement.

**TABLE 2:** Random and fixed effects regression estimates for out-of-school suspension.

	M1 random effects	M2 random effects with district fixed effects	M3 child fixed effects
	OR (95% CI)	OR (95% CI)	OR (95% CI)
CPS contact in the past and upcoming 6 months (reference = no CPS)			
Recent or current CPS investigation, no foster	1.78 (1.63–1.95)***	1.76 (1.61–1.92)***	1.49 (1.39–1.59)***
Recent or current foster care (CPS-FC)	1.17 (0.97–1.40)	1.18 (0.98–1.42)	1.05 (0.93–1.19)
Future CPS—Any CPS contact in next 6 months, none in past 6 months	1.60 (1.46–1.76)***	1.59 (1.45–1.74)***	1.33 (1.24–1.44)***
CPS involvement prior to kindergarten (reference = none)			
1 CPS report, NP	2.11 (1.93–2.30)***	2.09 (1.91–2.28)***	—
Multiple CPS reports, NP	3.01 (2.69–3.36)***	3.00 (2.68–3.35)***	—
FC to reunification	2.43 (2.02–2.92)***	2.35 (1.96–2.82)***	—
FC to adoption	1.59 (1.15–2.21)**	1.61 (1.15–2.25)**	—
FC to other	2.94 (2.37–3.63)***	2.91 (2.34–3.62)***	—
Demographics			
Male	2.66 (2.50–2.83)***	2.68 (2.52–2.85)***	—
Black	3.67 (3.32–4.07)***	3.43 (3.11–3.79)***	—
Hispanic	1.20 (1.09–1.32)***	1.15 (1.05–1.27)**	—
Native American	1.88 (1.54–2.29)***	2.02 (1.62–2.51)***	—
Asian/Pacific islander	0.36 (0.28–0.46)***	0.32 (0.25–0.41)***	—
Multiracial	2.38 (2.10–2.70)***	2.15 (1.89–2.44)***	—
Old for grade level	1.07 (0.99–1.17)	1.08 (0.99–1.18)	1.13 (1.01–1.28)*
Past year special education disability (reference = none)			
Autism	1.45 (1.18–1.78)***	1.46 (1.19–1.79)***	0.72 (0.59–0.88)**
Learning disability	1.39 (1.22–1.58)***	1.43 (1.26–1.63)***	1.19 (1.04–1.38)*
Speech & language	0.93 (0.78–1.11)	0.90 (0.76–1.08)	0.88 (0.77–1.01)
Intellectual disability/traumatic brain injury	1.14 (0.88–1.48)	1.14 (0.87–1.48)	0.97 (0.75–1.26)
Emotional behavioral	2.76 (2.40–3.16)***	2.85 (2.48–3.28)***	1.19 (1.09–1.29)***
Physical impairment	0.95 (0.59–1.53)	0.97 (0.60–1.57)	0.56 (0.31–1.03)
Significant developmental delay	1.67 (1.16–2.39)**	2.00 (1.41–2.84)***	1.22 (0.92–1.61)
Other	1.79 (1.60–2.00)***	1.79 (1.59–2.02)***	1.17 (1.06–1.28)**
Prior SY homelessness	1.36 (1.23–1.51)***	1.34 (1.21–1.48)***	1.21 (1.13–1.31)***
Prior SY free or reduced lunch	1.90 (1.77–2.03)***	1.90 (1.78–2.03)***	1.14 (1.07–1.22)***
Additional covariates/specifications			
Grade level fixed effects	Yes	Yes	Yes
Calendar month fixed effects	Yes	Yes	Yes
Prior suspensions covariate	Yes	Yes	No
Time-varying district covariates	Yes	No	Yes
District fixed effects	No	Yes	No
Child fixed effects	No	No	Yes
*N* Children	49,918	48,380	6429
*N* Child-Months	4.3 M	4.1 M	540,018

*Note*: M1 = random effects model; M2 = random effects model with school district fixed effects; M3 = within-child fixed effects.

*Note*: Reference for male is female; reference group for race and ethnicity is non-Hispanic white.

Abbreviations: CPS, child protective services; FC, foster care; NP, no placement; SY, school year.

* *p* < .05

** *p* < .01

*** *p* < .001.

**TABLE 3:** Subgroup models by grade level and current school year special education status.

	Random effects with district fixed effects (M2)	Child fixed effects (M3)
	OR (95% CI)	OR (95% CI)
Kindergarten through fourth grade		
CPS involvement in the past and upcoming 6 months (reference = no CPS)		
Recent or current CPS-NP	1.57 (1.40–1.77)***	1.26 (1.12–1.41)***
Recent or current CPS-FC	1.13 (0.89–1.43)	0.94 (0.73–1.20)
Future CPS (no recent or current)	1.53 (1.35–1.73)***	1.22 (1.08–1.38)**
*N* Children	42,599	2516
*N* Child-Months	2,045,708	121,278
Fifth-sixth grade		
CPS involvement in the past and upcoming 6 months (reference = no CPS)		
Recent or current CPS-NP	2.03 (1.75–2.35)***	1.54 (1.30–1.82)***
Recent or current CPS-FC	1.05 (0.79–1.41)	0.78 (0.52–1.17)
Future CPS (no recent or current)	1.72 (1.47–2.02)***	1.24 (1.04–1.47)*
*N* Children	42,502	2599
*N* Child-Months	787,902	47,747
Seventh—Eighth Grade		
CPS involvement in the past and upcoming 6 months (reference = no CPS)		
Recent or current CPS-NP	1.89 (1.67–2.14)***	1.37 (1.19–1.58)***
Recent or current CPS-FC	1.27 (1.03–1.56)*	0.88 (0.65–1.20)
Future CPS (no recent or current)	1.58 (1.38–1.81)***	1.10 (0.95–1.28)
*N* Children	45,807	4500
*N* Child-Months	807,154	78,496
No special education placement		
CPS involvement in the past and upcoming 6 months (reference = no CPS)		
Recent or current CPS-NP	1.84 (1.66–2.04)***	1.46 (1.32–1.62)***
Recent or current CPS-FC	1.70 (1.39–2.07)***	1.35 (1.09–1.67)**
Future CPS (no recent or current)	1.63 (1.46–1.82)***	1.28 (1.15–1.43)***
*N* Children	44,938	4788
*N* Child-Months	3,472,187	365,101
Special education placement during school year		
CPS involvement in the past and upcoming 6 months (reference = no CPS)		
Recent or current CPS-NP	1.69 (1.54–1.86)***	1.43 (1.30–1.57)***
Recent or current CPS-FC	1.12 (0.95–1.31)	0.90 (0.76–1.07)
Future CPS (no recent or current)	1.54 (1.39–1.70)***	1.28 (1.15–1.41)***
*N* Children	10,311	2196
*N* Child-Months	511,954	133,428

*Note*: Outcome is any suspension within child-month. Subgroup random effects model has identical specifications and covariates as Model 2 of Table 2 and Subgroup child fixed effects model has identical specifications and covariates as Model 3 of Table 2.

Abbreviations: CPS, child protective services; FC, foster care; NP, no placement.

* *p* < .05

** *p* < .01

*** *p* < .001.

**TABLE 4:** Subgroup models by sex and racial-ethnic category.

	Random effects with district fixed effects (M2)	Child fixed effects (M3)
	OR (95% CI)	OR (95% CI)
Male		
CPS involvement in the past and upcoming 6 months (reference = no CPS)		
Recent or current CPS-NP	1.68 (1.51–1.87)***	1.44 (1.33–1.56)***
Recent or current CPS-FC	1.10 (0.89–1.36)	1.02 (0.87–1.19)
Future CPS (no recent or current)	1.57 (1.40–1.75)***	1.33 (1.22–1.46)***
*N* Children	24,788	4477
*N* Child-months	2,087,175	376,476
Female		
CPS involvement in the past and upcoming 6 months (reference = no CPS)		
Recent or current CPS-NP	1.86 (1.58–2.18)***	1.47 (1.30–1.67)***
Recent or current CPS-FC	1.30 (0.89–1.89)	1.04 (0.83–1.30)
Future CPS (no recent or current)	1.57 (1.32–1.86)***	1.25 (1.09–1.43)**
*N* Children	20,836	1956
*N* Child-months	1,708,996	162,890
White		
CPS involvement in the past and upcoming 6 months (reference = no CPS)		
Recent or current CPS-NP	1.82 (1.56–2.13)***	1.50 (1.34–1.68)***
Recent or current CPS-FC	0.99 (0.70–1.42)	0.89 (0.72–1.10)
Future CPS (no recent or current)	1.82 (1.55–2.12)***	1.47 (1.30–1.66)***
*N* Children	35,641	3107
*N* Child-months	2,951,806	259,373
Black		
CPS involvement in the past and upcoming 6 months (reference = no CPS)		
Recent or current CPS-NP	1.64 (1.43–1.87)***	1.49 (1.34–1.66)***
Recent or current CPS-FC	1.31 (1.04–1.66)*	1.18 (0.98–1.43)
Future CPS (no recent or current)	1.46 (1.27–1.68)***	1.31 (1.17–1.47)***
*N* Children	3670	1809
*N* Child-months	276,493	145,099
Hispanic		
CPS involvement in the past and upcoming 6 months (reference = no CPS)		
Recent or current CPS-NP	2.19 (1.73–2.78)***	1.72 (1.43–2.07)***
Recent or current CPS-FC	1.21 (0.74–1.97)	0.80 (0.51–1.27)
Future CPS (no recent or current)	1.45 (1.12–1.89)**	1.15 (0.92–1.44)
*N* Children	5302	986
*N* Child-months	405,392	77,698

*Note*: Outcome is any suspension within child-month. Subgroup random effects model has identical specifications and covariates as Model 2 of Table 2 and Subgroup child fixed effects model has identical specifications and covariates as Model 3 of Table 2.

Abbreviations: CPS, child protective services; FC, foster carel; NP, no placement.

* *p* < .05

** *p* < .01

*** *p* < .001.

**TABLE 5:** Alternative specifications—source of cps report and detailed foster care status.

	Random effects with district fixed effects (M2)	Child fixed effects (M3)
	OR (95% CI)	OR (95% CI)
Source of report (outcome = rule violation suspensions)		
Version 1 – Rule Violations, all grades		
CPS contact within past 6 months (reference = none)	—	—
Recent or current CPS-NP, education report source	2.35 (2.04–2.69)***	1.63 (1.43–1.85)***
Recent or current CPS-NP, other report source	1.56 (1.41–1.71)***	1.26 (1.15–1.38)***
Recent or current CPS-FC	1.10 (0.94–1.27)	1.03 (0.89–1.20)
*N* Children	47,830	4887
*N* Child-months	4,035,254	409,634
Version 2 – Rule violations, K-4th only		
Past 6 months CPS contact (reference = none)	—	—
CPS from education report source	2.19 (1.75–2.73)***	1.58 (1.27–1.96)***
CPS from other report source	1.44 (1.21–1.72)***	1.14 (0.96–1.36)
CPS-FC	1.02 (0.77–1.35)	0.82 (0.61–1.12)
*N* Children	37,902	1710
*N* Child-months	1,790,026	82,415
Detailed foster care measure (outcome = any suspension)		
CPS involvement in the past and upcoming 6 months (reference = no CPS)		
Recent or current CPS-NP	1.76 (1.64–1.88)***	1.48 (1.39–1.59)***
Recent or current CPS-FC, new removal	1.36 (1.06–1.74)*	1.23 (0.97–1.57)
Recent or current CPS-FC, ongoing removal	1.06 (0.91–1.24)	0.93 (0.79–1.08)
Recent or current CPS-FC, no longer in care	1.35 (1.08–1.68)**	1.22 (0.98–1.52)
Future CPS (no recent or current)	1.59 (1.47–1.71)***	1.33 (1.24–1.43)***
*N* Children	48,380	6249
*N* Child-months	4,095,487	540,018

*Note*: Random effects model has identical specifications and covariates as Model 2 of Table 2 and Subgroup child fixed effects model has identical specifications and covariates as Model 3 of Table 2.

Abbreviations: CPS, child protective services; FC, foster care; NP, no placement.

* *p* < .05

** *p* < .01

*** *p* < .001.

## Data Availability

The data necessary to reproduce the analyses presented here are not publicly accessible. Inquiries about data access should be directed to the Institute for Research on Poverty at the University of Wisconsin-Madison. Other study materials, such as code, can be requested from the lead author. The analyses presented here were not preregistered.

## Abbreviations:

CPS: child protective services
DPI: Department of Public Instruction
FC: foster care
NP: no placement
WADC: Wisconsin Administrative Data Core

## References

[R1] Afifi TO, McTavish J, Turner S, MacMillan HL, & Wathen CN (2018). The relationship between child protection contact and mental health outcomes among Canadian adults with a child abuse history. Child Abuse & Neglect, 79, 22–30. 10.1016/j.chiabu.2018.01.019 29407853

[R2] Bacher-Hicks A, Billings S, & Deming D (2019). The school to prison pipeline: Long-run impacts of school suspensions on adult crime. (No. w26257; p. w26257). National Bureau of Economic Research. 10.3386/w26257

[R3] Bald A, Chyn E, Hastings JS, & Machelett M (2019). The causal impact of removing children from abusive and neglectful homes. National Bureau of Economic Research. https://www.nber.org/papers/w25419

[R4] Baron EJ, & Gross M (2022). Is there a foster care-to-prison pipeline? Evidence from quasi-randomly assigned investigators. (Working Paper No. 29922). National Bureau of Economic Research. 10.3386/w29922

[R5] Barrett N, McEachin A, Mills JN, & Valant J (2021). Disparities and discrimination in student discipline by race and family income. Journal of Human Resources, 56, 711–748. 10.3368/jhr.56.3.0118-9267R2

[R6] Barth RP, Jonson-Reid M, Greeson JKP, Drake B, Berrick JD, Garcia AR, Shaw TV, & Gyourko JR (2020). Outcomes following child welfare services: What are they and do they differ for black children? Journal of Public Child Welfare, 14, 477–499. 10.1080/15548732.2020.1814541

[R7] Bell MF, Bayliss DM, Glauert R, & Ohan JL (2018). School readiness of maltreated children: Associations of timing, type, and chronicity of maltreatment. Child Abuse & Neglect, 76, 426–439. 10.1016/j.chiabu.2017.12.001 29245140

[R8] Berger LM, Bruch SK, Johnson EI, James S, & Rubin D (2009). Estimating the “impact” of out-of-home placement on child well-being: Approaching the problem of selection bias. Child Development, 80, 1856–1876. 10.1111/j.1467-8624.2009.01372.x 19930356 PMC2836492

[R9] Berger LM, Cancian M, Han E, Noyes J, & Rios-Salas V (2015). Children's academic achievement and foster care. Pediatrics, 135, 109–116. 10.1542/peds.2014-2448 PMC7552920 25535267

[R10] Berger LM, & Font SA (2015). The role of the family and family-centered programs and policies. Future of Children, 25, 155–176. 10.1353/foc.2015.0007 30679897 PMC6342196

[R11] Braga T, Cunha O, & Maia Â (2018). The enduring effect of maltreatment on antisocial behavior: A meta-analysis of longitudinal studies. Aggression and Violent Behavior, 40, 91–100. 10.1016/j.avb.2018.04.003

[R12] Carr CL, McLeigh J, Roman H, Fults JB, Gonzalez JR, Sanders C, Clutter MO, Tsai R, & Jetelina KK (2020). Healthcare utilization patterns among children with a history of Child Protective Services investigations. Violence and Victims, 35, 906–919. 10.1891/VV-D-19-00122 33372116

[R13] Cho M, Haight W, Choi WS, Hong S, & Piescher K (2019). A prospective, longitudinal study of risk factors for early onset of delinquency among maltreated youth. Children and Youth Services Review, 102, 222–230. 10.1016/j.childyouth.2019.05.023

[R14] Crozier JC, & Barth RP (2005). Cognitive and academic functioning in maltreated children. Children & Schools, 27, 197–206. 10.1093/cs/27.4.197

[R15] Davison M, Penner AM, Penner EK, Pharris-Ciurej N, Porter SR, Rose EK, Shem-Tov Y, & Yoo P (2022). School discipline and racial disparities in early adulthood. Educational Researcher, 51, 231–234. 10.3102/0013189X211061732 35874270 PMC9307071

[R16] Dettlaff AJ, Weber K, Pendleton M, Boyd R, Bettencourt B, & Burton L (2020). It is not a broken system, it is a system that needs to be broken: The upEND movement to abolish the child welfare system. Journal of Public Child Welfare, 14, 500–517. 10.1080/15548732.2020.1814542

[R17] Doyle JJ Jr. (2007). Child protection and child outcomes: Measuring the effects of foster care. American Economic Review, 97, 1583–1610. 10.1257/aer.97.5.1583 29135212

[R18] Doyle JJ Jr. (2008). Child protection and adult crime: Using investigator assignment to estimate causal effects of foster care. Journal of Political Economy, 116, 746–770. 10.1086/590216

[R19] Fenelon A, Slopen N, & Newman SJ (2022). The effects of rental assistance programs on neighborhood outcomes for U.S. children: Nationwide evidence by program and race/ethnicity. Urban Affairs Review, in press, 59, 832–865. 10.1177/10780874221098376 PMC12124806 40453363

[R20] Folman RD (1998). “I was tooken” How children experience removal from their parents preliminary to placement into foster care. Adoption Quarterly, 2, 7–35. 10.1300/J145v02n02_02

[R21] Font SA, & Berger LM (2015). Child maltreatment and children's developmental trajectories in early to middle childhood. Child Development, 86, 536–556. 10.1111/cdev.12322 25521556 PMC4376662

[R22] Font SA, Caniglia M, Kennedy R, & Noll JG (2022). Child protection intervention and the sexual and reproductive health of female adolescents ages 13 to 17 years. JAMA Pediatrics, 176, 461–469. 10.1001/jamapediatrics.2021.6605 35188543 PMC8861893

[R23] Font SA, & Kennedy R (2022). The centrality of child maltreatment to criminology. Annual Review of Criminology, 5, 371–396. 10.1146/annurev-criminol-030920-120220 PMC9216336 35756098

[R24] Font SA, & Maguire-Jack K (2013). Academic engagement and performance: Estimating the impact of out-of-home care for maltreated children. Children and Youth Services Review, 35, 856–864.

[R25] Font SA, Miyamoto S, & Pinto C (2020). Child sexual abuse and exploitation in Pennsylvania. Center for Rural Pennsylvania, 1–120. https://www.rural.palegislature.us/documents/reports/Child-Sexual-Abuse-and-Exploitation-2020.pdf

[R26] Font SA, & Palmer L (2022). Timely permanency for children in foster care: Revisiting core assumptions about children's options and outcomes. Court Review, 58, 26–32.

[R27] Garland AF, Landsverk JA, & Lau AS (2003). Racial/ethnic disparities in mental health service use among children in foster care. Children and Youth Services Review, 25, 491–507.

[R28] Government Accountability Office. (2015). HHS could do more to support states' efforts to keep children in family-based care (GAO-16-85). https://www.gao.gov/assets/gao-16-85.pdf

[R29] Gross M, & Baron EJ (2022). Temporary stays and persistent gains: The causal effects of foster care. American Economic Journal: Applied Economics, 14, 170–199. 10.1257/app.20200204

[R30] Harden BJ, Buhler A, & Parra LJ (2016). Maltreatment in infancy: A developmental perspective on prevention and intervention. Trauma, Violence, & Abuse, 17, 366–386. 10.1177/1524838016658878 27580663

[R31] Harman JS, Childs GE, & Kelleher KJ (2000). Mental health care utilization and expenditures by children in foster care. Archives of Pediatrics & Adolescent Medicine, 154, 1114–1117. 10.1001/archpedi.154.11.1114 11074852

[R32] Helton JJ, Vaughn MG, & Schiff M (2022). The accrual of parent reported adverse childhood experiences following a child protective services investigation: A prospective approach. Child Abuse & Neglect, 124, 105447. 10.1016/j.chiabu.2021.105447 34923299

[R33] Herrenkohl TI, & Herrenkohl RC (2007). Examining the overlap and prediction of multiple forms of child maltreatment, stressors, and socioeconomic status: A longitudinal analysis of youth outcomes. Journal of Family Violence, 22, 553–562.

[R34] Holmes MR, Yoon S, Berg KA, Cage JL, & Perzynski AT (2018). Promoting the development of resilient academic functioning in maltreated children. Child Abuse & Neglect, 75, 92–103. 10.1016/j.chiabu.2017.07.018 28784310

[R35] Iceland J, Weinberg DH, & Steinmetz E (2002). Racial and ethnic residential segregation in the United States: 1980-2000. https://www.census.gov/prod/2002pubs/censr-3.pdf

[R36] Ireland TO, Smith CA, & Thornberry TP (2002). Developmental issues in the impact of child maltreatment on later delinquency and drug use. Criminology, 40, 359–400.

[R37] Jones LM, Cross TP, Walsh WA, & Simone M (2007). Do Children's Advocacy Centers improve families' experiences of child sexual abuse investigations? Child Abuse & Neglect, 31, 1069–1085. 10.1016/j.chiabu.2007.07.003 17997155

[R38] Jonson-Reid M, Drake B, Kohl P, Guo S, Brown D, McBride T, Kim H, & Lewis E (2017). What do we really know about usual care child protective services? Children and Youth Services Review, 82, 222–229. 10.1016/j.childyouth.2017.09.019

[R39] Kim H, & Drake B (2019). Cumulative prevalence of onset and recurrence of child maltreatment reports. Journal of the American Academy of Child & Adolescent Psychiatry, 58(12), 1175–1183. 10.1016/j.jaac.2019.02.015 30851398

[R40] Konijn C, Admiraal S, Baart J, van Rooij F, Stams G-J, Colonnesi C, Lindauer R, & Assink M (2019). Foster care placement instability: A meta-analytic review. Children and Youth Services Review, 96, 483–499. 10.1016/j.childyouth.2018.12.002

[R41] Lazebnik R, Zimet GD, Ebert J, Anglin TM, Williams P, Bunch DL, & Krowchuk DP (1994). How children perceive the medical evaluation for suspected sexual abuse. Child Abuse & Neglect, 18, 739–745. 10.1016/0145-2134(94)00040-9 8000904

[R42] Leslie LK, Hurlburt MS, Landsverk J, Barth R, & Slymen DJ (2004). Outpatient mental health services for children in foster care: A national perspective. Child Abuse & Neglect, 28, 697–712. 10.1016/j.chiabu.2004.01.004 15193856

[R43] Loomis AM, Feely M, & Kennedy S (2020). Measuring self-reported polyvictimization in foster youth research: A systematic review. Child Abuse & Neglect, 107, 104588. 10.1016/j.chiabu.2020.104588 32535337

[R44] Manly JT, Lynch M, Oshri A, Herzog M, & Wortel SN (2013). The Impact of neglect on initial adaptation to school. Child Maltreatment, 18, 155–170. 10.1177/1077559513496144 23843472 PMC3775317

[R45] Masson M, Bussières E-L, East-Richard C, R-Mercier A, & Cellard C (2015). Neuropsychological profile of children, adolescents and adults experiencing maltreatment: A meta-analysis. The Clinical Neuropsychologist, 29, 573–594. 10.1080/13854046.2015.1061057 26114949

[R46] McFarlane K (2018). Care-criminalisation: The involvement of children in out-of-home care in the New South Wales criminal justice system. Australian & New Zealand Journal of Criminology, 51, 412–433. 10.1177/0004865817723954

[R47] McGuire A, & Jackson Y (2018). Dimensions of maltreatment and academic outcomes for youth in foster care. Child Abuse & Neglect, 84, 82–94. 10.1016/j.chiabu.2018.07.029 30071396 PMC11364216

[R48] McKenna S, Donnelly M, Onyeka IN, O'Reilly D, & Maguire A (2021). Experience of child welfare services and long-term adult mental health outcomes: A scoping review. Social Psychiatry and Psychiatric Epidemiology, 56, 1115–1145. 10.1007/s00127-021-02069-x 33779782 PMC8225538

[R49] McMillen JC, Scott LD, Zima BT, Ollie MT, Munson MR, & Spitznagel E (2004). Use of mental health services among older youths in foster care. Psychiatric Services, 55, 811–817. 10.1176/appi.ps.55.7.811 15232022

[R50] McMillen JC, Zima BT, Scott LD, Auslander W, Munson M, Ollie M, & Spitznagel E (2005). Prevalence of psychiatric disorders among older youths in the foster care system. Journal of the American Academy of Child & Adolescent Psychiatry, 44, 88–95. 10.1097/01.chi.0000145806.24274.d2 15608548

[R51] Messman-Moore TL, & Bhuptani PH (2017). A review of the long-term impact of child maltreatment on posttraumatic stress disorder and its comorbidities: an emotion dysregulation perspective. Clinical Psychology: Science and Practice, 24, 154–169. 10.1111/cpsp.12193

[R52] Mitchell MB (2018). “No one acknowledged my loss and hurt”: Non-death loss, grief, and trauma in foster care. Child and Adolescent Social Work Journal, 35, 1–9. 10.1007/s10560-017-0502-8

[R53] Mittleman J (2018). A downward spiral? Childhood suspension and the path to juvenile arrest. Sociology of Education, 91, 183–204. 10.1177/0038040718784603

[R54] Noltemeyer AL, Ward RM, & Mcloughlin C (2015). Relationship between school suspension and student outcomes: A meta-analysis. School Psychology Review, 44, 224–240. 10.17105/spr-14-0008.1

[R55] Owens A (2018). Income segregation between school districts and inequality in students' achievement. Sociology of Education, 91, 1–27. 10.1177/0038040717741180

[R56] Palmer L, Ahn E, Traube D, Prindle J, & Putnam-Hornstein E (2020). Correlates of entry into congregate care among a cohort of California foster youth. Children and Youth Services Review, 110, 1–6. 10.1016/j.childyouth.2020.104772

[R57] Palmer L, Font S, Herd T, Prindle J, & Putnam-Hornstein E (2022). Rates of emotional disturbance among children in foster care: Comparing federal child welfare data and Medicaid records in two states. Child Maltreatment, in press, 107755952211189. 10.1177/10775595221118931 PMC10367964 35950631

[R58] Raz M (2020). Calling child protectives services is a form of community policing that should be used appropriately: Time to engage mandatory reporters as to the harmful effects of unnecessary reports. Children and Youth Services Review, 110, 1–4. 10.1016/j.childyouth.2020.104817

[R59] Roberts D (2021). Torn apart: How the child welfare system destroys Black families—and how abolition can build a safer world. Basic Books. https://www.basicbooks.com/titles/dorothy-roberts/torn-apart/9781549193170/

[R60] Roberts K (2019). Foster care and child welfare. [Dissertation, Clemson University]. https://tigerprints.clemson.edu/all_dissertations/2375

[R61] Sariaslan A, Kääriälä A, Pitkänen J, Remes H, Aaltonen M, Hiilamo H, Martikainen P, & Fazel S (2022). Long-term health and social outcomes in children and adolescents placed in out-of-home care. JAMA Pediatrics, 176, e214324. 10.1001/jamapediatrics.2021.4324 34694331 PMC8546624

[R62] Scherr TG (2007). Educational experiences of children in foster care: Meta-analyses of special education, retention and discipline rates. School Psychology International, 28, 419–436. 10.1177/0143034307084133

[R63] Shenk CE, Noll JG, Peugh JL, Griffin AM, & Bensman HE (2016). Contamination in the prospective study of child maltreatment and female adolescent health. Journal of Pediatric Psychology, 41, 37–45. 25797944 10.1093/jpepsy/jsv017 PMC4710181

[R64] Skiba RJ, Chung C-G, Trachok M, Baker TL, Sheya A, & Hughes RL (2014). Parsing disciplinary disproportionality: Contributions of infraction, student, and school characteristics to out-of-school suspension and expulsion. American Educational Research Journal, 51, 640–670. 10.3102/0002831214541670

[R65] Spratt EG, Friedenberg SL, Swenson CC, LaRosa A, De Bellis MD, Macias MM, Summer AP, Hulsey TC, Runyan DK, & Brady KT (2012). The effects of early neglect on cognitive, language, and behavioral functioning in childhood. Psychology, 3, 175–182. 10.4236/psych.2012.32026 23678396 PMC3652241

[R66] Szilagyi MA, Rosen DS, Rubin D, & Zlotnik S (2015). Health care issues for children and adolescents in foster care and kinship care. Pediatrics, 136, e1142–e1166. 26416934 10.1542/peds.2015-2656

[R67] U.S. Department of Health and Human Services. (2020). The AFCARS report: Preliminary FY 2019 estimates as of June 23, 2020. (No. 27). https://www.acf.hhs.gov/sites/default/files/documents/cb/afcarsreport27.pdf

[R68] U.S. Department of Health and Human Services. (2021). Child maltreatment 2019. Administration for Children and Families. https://www.acf.hhs.gov/sites/default/files/documents/cb/cm2019.pdf

[R69] U.S. General Accounting Office. (1998). Foster care: Agencies face challenges securing stable homes for children of substance abusers (GAO/HEHS-98-182). https://www.gao.gov/assets/230/226295.pdf

[R70] Valdebenito S, Eisner M, Farrington DP, Ttofi MM, & Sutherland A (2018). School-based interventions for reducing disciplinary school exclusion: A systematic review. Campbell Systematic Reviews, 14, 1–216. 10.4073/csr.2018.1 PMC8533648 37131379

[R71] Veltman MWM, & Browne KD (2001). Three decades of child maltreatment research: Implications for the school years. Trauma, Violence, & Abuse, 2, 215–239. 10.1177/1524838001002003002

[R72] Welsh RO, & Little S (2018). The school discipline dilemma: A comprehensive review of disparities and alternative approaches. Review of Educational Research, 88, 752–794. 10.3102/0034654318791582

[R73] Wildeman C, Scardamalia K, Walsh EG, O'Brien RL, & Brew B (2017). Paternal incarceration and teachers' expectations of students. Socius, 3. 10.1177/2378023117726610

[R74] Wilson S, Hean S, Abebe T, & Heaslip V (2020). Children's experiences with Child Protection Services: A synthesis of qualitative evidence. Children and Youth Services Review, 113, 104974. 10.1016/j.childyouth.2020.1049744

