# Supplementary material for: Child protective services involvement and exclusionary school discipline
Source: Child Dev. Author manuscript; Available in PMC 2024 Feb 16. (PMC10636238; doi:10.1111/cdev.13941)
Supplement: supplement [file NIHMS1911545-supplement-supplement.docx]

| Appendix A.  *Estimated Effects of Any CPS Involvement on Suspension* | | | | | | |
| --- | --- | --- | --- | --- | --- | --- |
|  | M1  Random effects | | M2  Random effects with school district fixed effects | | M3  Child fixed effects | |
|  | OR | 95% CI | OR | 95% CI | OR | 95% CI |
| CPS involvement in the past and upcoming six months  (reference = none) | |  |  |  |  |  |
| Recent or current CPS | 1.62*** | (1.49-1.77) | 1.61*** | (1.48-1.76) | 1.38*** | (1.30-1.47) |
| Future CPS (no recent or current) | 1.60*** | (1.46-1.76) | 1.59*** | (1.45-1.75) | 1.34*** | (1.24-1.44) |
| **CPS involvement prior to kindergarten** (reference = none) | | | |  |  |  |
| None |  |  |  |  |  |  |
| 1 CPS report | 2.11*** | (1.93-2.31) | 2.09*** | (1.91-2.29) |  | - |
| Multiple CPS reports | 3.00*** | (2.69-3.36) | 2.99*** | (2.68-3.34) |  | - |
| FC reunified | 2.42*** | (2.01-2.91) | 2.34*** | (1.95-2.81) |  | - |
| FC adopted | 1.59** | (1.15-2.21) | 1.61** | (1.15-2.25) |  | - |
| FC other | 2.81*** | (2.27-3.48) | 2.79*** | (2.25-3.46) |  | - |
| **Demographics** |  |  |  |  |  |  |
| Male | 3.68*** | (3.32-4.07) | 3.44*** | (3.11-3.80) |  | - |
| Black | 1.20*** | (1.09-1.33) | 1.16** | (1.05-1.27) |  | - |
| Hispanic | 1.88*** | (1.53-2.29) | 2.01*** | (1.61-2.51) |  | - |
| Native American | 0.36*** | (0.28-0.46) | 0.32*** | (0.25-0.41) |  | - |
| Asian/Pacific islander | 2.39*** | (2.11-2.71) | 2.16*** | (1.90-2.45) |  | - |
| Multiracial | 1.07 | (0.98-1.16) | 1.08 | (0.99-1.17) |  | - |
| Old for grade level | 3.68*** | (3.32-4.07) | 3.44*** | (3.11-3.80) | 1.13* | (1.01-1.27) |
| **Past year special education disability** (reference = none) | |  |  |  |  | 1.13* |
| Autism | 1.45*** | (1.18-1.78) | 1.46*** | (1.19-1.80) | 0.72** | (0.59-0.88) |
| Learning disability | 1.39*** | (1.22-1.58) | 1.43*** | (1.26-1.63) | 1.19* | (1.03-1.38) |
| Speech & language | 0.93 | (0.78-1.11) | 0.91 | (0.76-1.08) | 0.88 | (0.77-1.02) |
| Intellectual disability/traumatic brain injury | 1.14 | (0.88-1.48) | 1.14 | (0.87-1.48) | 0.97 | (0.75-1.26) |
| Emotional behavioral | 2.73*** | (2.38-3.13) | 2.83*** | (2.46-3.25) | 1.18*** | (1.09-1.28) |
| Physical impairment | 0.95 | (0.59-1.53) | 0.97 | (0.60-1.57) | 0.56 | (0.31-1.03) |
| Significant developmental delay | 1.67** | (1.17-2.40) | 2.01*** | (1.42-2.85) | 1.23 | (0.93-1.62) |
| Other | 1.79*** | (1.60-2.01) | 1.79*** | (1.59-2.02) | 1.17** | (1.06-1.29) |
| Prior SY homelessness | 1.36*** | (1.23-1.51) | 1.34*** | (1.21-1.48) | 1.22*** | (1.13-1.31) |
| Prior SY free/reduced lunch | 1.89*** | (1.76-2.02) | 1.89*** | (1.77-2.02) | 1.14*** | (1.07-1.21) |
| **Additional Covariates/Specifications** | |  |  |  |  |  |
| Grade level fixed effects | Yes | | Yes | | Yes | |
| Calendar month fixed effects | Yes | | Yes | | Yes | |
| Prior suspensions covariate | Yes | | Yes | | No | |
| Time-varying district covariates | Yes | | No | | Yes | |
| District fixed effects | No | | Yes | | No | |
| Child fixed effects | No | | No | | Yes | |
| *N* children | 49,918 | | 48,380 | | 6,429 | |
| *N* child months | 4.3M | | 4.1M | | 540,018 | |

Notes: CPS=child protective services; NP=no placement; FC=foster care; SY=school year. Reference for male is female; reference group for race/ethnicity is non-Hispanic white. M1=random effects model; M2= random effects model with school district fixed effects; M3=within-child fixed effects.

**p*<.05, ***p*<.01, ****p*<.001

| Appendix B.  *Random and Fixed Effects Linear Regression Estimates for Out-of-School Suspension* | | | | | | |  |  |
| --- | --- | --- | --- | --- | --- | --- | --- | --- |
|  | M1  Random effects | | M2  Random effects with school district fixed effects | | M3  Child fixed effects, full sample | | M4  Child fixed effects, logit subsample | |
|  | b | 95% CI | b | 95% CI | b | 95% CI | b | 95% CI |
| **CPS contact in the past and upcoming six months** (reference=no CPS) | |  |  |  |  |  |  |  |
| Recent or current CPS-NP | 0.76*** | (0.58-0.93) | 0.79*** | (0.61-0.96) | 0.57*** | (0.51-0.63) | 1.69*** | (1.41-1.97) |
| Recent or current CPS-FC | 0.21 | (-0.17-0.58) | 0.24 | (-0.14-0.62) | 0.22*** | (0.10-0.34) | 0.35 | (-0.15-0.85) |
| Future CPS (no recent or current) | 0.53*** | (0.37-0.68) | 0.55*** | (0.39-0.70) | 0.31*** | (0.25-0.38) | 1.09*** | (0.80-1.39) |
| **CPS involvement prior to kindergarten** (reference = none) | | | |  |  |  |  |  |
| 1 CPS report, NP | 0.16*** | (0.08-0.24) | 0.17*** | (0.09-0.25) | - | - | - | - |
| Multiple CPS reports, NP | 0.43*** | (0.27-0.58) | 0.44*** | (0.29-0.60) | - | - | - | - |
| FC to reunification | 0.06 | (-0.17-0.29) | 0.07 | (-0.16-0.30) | - | - | - | - |
| FC to adoption | -0.22* | (-0.42--0.02) | -0.24* | (-0.45--0.04) | - | - | - | - |
| FC to other | 0.32 | (-0.03-0.67) | 0.32 | (-0.04-0.67) | - | - | - | - |
| **Demographics** | - | - | - | - | - | - | - | - |
| Male | 0.11*** | (0.08-0.13) | 0.11*** | (0.09-0.14) | - | - | - | - |
| Black | 0.84*** | (0.72-0.95) | 0.82*** | (0.71-0.94) | - | - | - | - |
| Hispanic | -0.04 | (-0.09-0.01) | -0.03 | (-0.08-0.02) | - | - | - | - |
| Native American | 0.03 | (-0.10-0.16) | 0.12 | (-0.03-0.28) | - | - | - | - |
| Asian/Pacific islander | -0.12*** | (-0.15--0.09) | -0.13*** | (-0.16--0.09) | - | - | - | - |
| Multiracial | 0.21*** | (0.11-0.31) | 0.22*** | (0.12-0.32) | - | - | - | - |
| Old for grade level | 0.06* | (0.01-0.11) | 0.05* | (0.00-0.11) | 0.20*** | (0.13-0.27) | 0.82*** | (0.41-1.23) |
| **Past year special education disability** (reference = none) | |  |  |  |  |  |  |  |
| Autism | 0.11** | (0.04-0.19) | 0.12** | (0.03-0.20) | 0.31*** | (0.24-0.37) | 0.61** | (0.20-1.02) |
| Learning disability | 0.02 | (-0.02-0.06) | 0.02 | (-0.02-0.06) | 0.15*** | (0.10-0.19) | 0.41* | (0.07-0.76) |
| Speech & language | -0.03 | (-0.25-0.18) | -0.05 | (-0.27-0.17) | 0.89*** | (0.73-1.05) | 1.53** | (0.60-2.47) |
| Intellectual disability/traumatic brain injury | 2.30*** | (1.93-2.67) | 2.37*** | (1.99-2.74) | 3.94*** | (3.84-4.03) | 3.73*** | (3.41-4.05) |
| Emotional behavioral | 0.04 | (-0.23-0.30) | 0.03 | (-0.24-0.31) | 0.29* | (0.04-0.55) | -0.22 | (-2.07-1.64) |
| Physical impairment | 0.09 | (-0.09-0.28) | 0.15 | (-0.04-0.34) | 0.12 | (-0.01-0.26) | 0.07 | (-0.71-0.85) |
| Significant developmental delay | 0.67*** | (0.50-0.85) | 0.67*** | (0.49-0.86) | 1.44*** | (1.37-1.52) | 2.32*** | (1.97-2.68) |
| Other | 0.11** | (0.04-0.19) | 0.12** | (0.03-0.20) | 0.31*** | (0.24-0.37) | 0.61** | (0.20-1.02) |
|  |  |  |  |  |  |  |  |  |
| Prior SY homelessness | 0.61*** | (0.39-0.82) | 0.62*** | (0.40-0.84) | 0.50*** | (0.43-0.57) | 0.90*** | (0.59-1.21) |
| Prior SY free/reduced lunch | 0.09*** | (0.07-0.12) | 0.10*** | (0.07-0.12) | 0.12*** | (0.09-0.15) | 0.34*** | (0.16-0.52) |
| **Additional Covariates/Specifications** | |  |  |  |  |  |  |  |
| Grade level fixed effects | Yes | | Yes | | Yes | | Yes | |
| Calendar month fixed effects | Yes | | Yes | | Yes | | Yes | |
| Prior suspensions covariate | Yes | | Yes | | No | | No | |
| Time-varying district covariates | Yes | | No | | Yes | | Yes | |
| District fixed effects | No | | Yes | | No | | No | |
| Child fixed effects | No | | No | | Yes | | Yes | |
| *N* Children | 49918 | | 48587 | | 49918 | | 6553 | |
| *N* Child months | 4267908 | | 4117234 | | 4267908 | | 550351 | |
| Notes: Coefficients are interpreted as percentage point changes (outcome scaled as 100/0 instead of 1/0). CPS=child protective services; NP=no placement; FC=foster care; SY=school year. Reference for male is female; reference group for race/ethnicity is non-Hispanic white. M1=random effects model; M2= random effects model with school district fixed effects; M3=within-child fixed effects. **p*<.05, ***p*<.01, ****p*<.001 | | | | | | | | |

| Appendix C  *Cumulative Rates of CPS and Suspension by Subgroup (Percentages)* | | | |
| --- | --- | --- | --- |
|  | Suspended | Any CPS | CPS-FC |
| Sex |  |  |  |
| Female | 8.23 | 13.07 | 2.02 |
| Male | 17.72 | 12.50 | 2.12 |
| Race/ethnicity |  |  |  |
| White | 8.50 | 10.22 | 1.50 |
| Black | 51.71 | 28.57 | 6.07 |
| Hispanic | 17.56 | 16.51 | 2.16 |
| Native American | 26.71 | 26.88 | 8.39 |
| Asian/PacIsl | 4.51 | 8.57 | 0.86 |
| Multiracial | 23.40 | 22.51 | 4.87 |
|  |  |  |  |
| Grade level group |  |  |  |
| K-4th | 5.07 | 10.85 | 1.50 |
| 5th-6th | 5.29 | 4.80 | 0.74 |
| 7th-8th | 9.31 | 4.46 | 0.89 |
| Special Education |  |  |  |
| No | 10.31 | 11.85 | 1.66 |
| Yes | 20.11 | 20.20 | 4.00 |
| CPS = Child protective services; FC=foster care. Grade descriptives include 1 observation per student per grade group. Special education descriptives include 1 observation per student per special education status. | | | |

| Appendix D  *Differentiation of Type of Disciplinary Event* | | |  |  |
| --- | --- | --- | --- | --- |
|  | Random effects with school district fixed effects (M2) | | Child fixed effects (M3) | |
|  | OR | 95% CI | OR | 95% CI |
| **Severe Infractions** | | |  |  |
| CPS involvement in the past and upcoming six months  (reference=no CPS) |  |  |  |  |
| Recent or current CPS-NP | 1.63*** | (1.44-1.85) | 1.33*** | (1.20-1.47) |
| Recent or current CPS-FC | 1.29* | (1.04-1.59) | 1.14 | (0.95-1.37) |
| Future CPS (no recent or current) | 1.52*** | (1.34-1.73) | 1.22*** | (1.09-1.36) |
| *N* Children | 45958 |  | 3799 |  |
| *N* Child-months | 3836919 |  | 318270 |  |
| **Other Rule Violations** | |  |  |  |
| CPS involvement in the past and upcoming six months  (reference=no CPS) |  |  |  |  |
| Recent or current CPS-NP | 1.91*** | (1.72-2.11) | 1.56*** | (1.43-1.69) |
| Recent or current CPS-FC | 1.21 | (0.98-1.49) | 1.03 | (0.88-1.20) |
| Future CPS (no recent or current) | 1.71*** | (1.53-1.91) | 1.39*** | (1.27-1.52) |
| *N* Children | 47830 |  | 4887 |  |
| *N* Child-months | 4035254 |  | 409634 |  |

Note: Severe infractions include specific subcategories for infractions related to: drugs, alcohol, weapons, assault and endangerment. The origin data collapses all other rule violations into a single and undifferentiated category. : CPS=child protective services; NP=no placement; FC=foster care.
